# Annealing effect of thermotropic liquid crystalline copolyester fibers on thermo-mechanical properties and morphology

**DOI:** 10.1038/s41598-022-17431-5

**Published:** 2022-07-30

**Authors:** Sanghyeon Park, Yeji Na, A Young Kim, Lee Ku Kwac, Hong Gun Kim, Jin-Hae Chang

**Affiliations:** 1 Graduate School of Carbon Convergence Engineering, Jeonju University, Jeonju, 55069 South Korea; 2 Institute of Carbon Technology, Jeonju University, Jeonju, 55069 South Korea

**Keywords:** Engineering, Materials science

## Abstract

A series of thermotropic liquid crystal copolyesters (Co-TLCPs) was prepared by melt polymerization using 2,5-diethoxyterephthalic acid (DTA), 2,7-dihydroxynaphthalene (DHN), and *p*-hydroxybenzoic acid (HBA) monomers, where the HBA content was varied (0–5 mol). At 3 mol HBA, the Co-TLCPs formed nematic mesophases, while below this concentration, the liquid crystalline phase did not appear. The Co-TLCP sample with 3 mol HBA was subjected to melt spinning and heat-treated under various conditions (temperature and time) to investigate their effect on the thermo–mechanical properties and degree of crystallinity. The objective was to determine the critical heat treatment condition that can maximize the properties of the spun Co-TLCP fibers. The microstructure of the heat-treated fiber was investigated using scanning electron microscopy, and the optimal annealing conditions were confirmed based on the morphology of the fiber, which exhibited a skin–core structure owing to the varying heat and pressure conditions applied during spinning.

## Introduction

Thermotropic liquid crystal polymer (TLCP) is attracting significant attention owing to its application as an ultrahigh-strength fiber. Because of its high strength and elasticity, excellent heat and chemical resistance, low molding shrinkage, and small coefficient of linear expansion during processing, TLCPs are being employed in various applications, including high-performance fibers, engineering plastics, and polymer composites. In addition, melt blending of general-purpose thermoplastic resin and TLCP is currently being extensively studied because it can improve the strength and elasticity of polymer composite materials, as well as afford excellent processability and high performance^1,2^.

A wholly aromatic TLCP exhibits excellent mechanical properties, heat resistance, dimensional stability, and chemical resistance. Most TLCPs are composed of rigid-rod wholly aromatic monomers in the main chain^3–5^. Some commonly used monomers include terephthalic acid, hydroquinone, 4,4′-biphenol, and *p*-hydroxybenzoic acid (HBA), which impart good physical and thermo-mechanical properties in the TLCPs. Alternatively, TLCPs composed of monomers such as 6-hydroxynaphthoic acid (HNA), naphthalene diol derivatives, and naphthalene dicarboxylic acid isomers, in which the basic structure of the main chain contains a para-substituted carboxyl group or a benzene ring, exhibit high meting temperatures of approximately 600 °C^6–8^.

Although rigid rod-type TLCPs show excellent thermal and mechanical properties, they are difficult to process or exhibit extremely low solubility in common solvents owing to their rigid rod-type structure. To compensate for these shortcomings, studies on various methods and structures other than linear aromatic esters as mesogens are being conducted^9–11^. One such method is that of lowering the melting point by introducing a flexible alkyl or alkoxy structure into the rigid main chain to prevent molecular chain packing. Although the increased molecular width and distance between the molecular chains presents the problem of low isotropic temperatures, the resultant TLCP can be easily melt-processed using an appropriate substituent. Alternatively, a monomer with a bent structure instead of a rigid linear structure is introduced into the main chain to disrupt the perfect straight rod-shaped structure. This can also be achieved by introducing an asymmetric structure into the main chain or using a bulky substituent as a side group. Reportedly, it is possible to melt or injection mold the TLCP below 400 °C using monomers containing flexible alkyl or alkoxy groups, bulk substituents, monomers with asymmetric structures, and branched or meta-substituted monomers, suggesting improved processability^3,9,11^. However, these TLCPs often exhibit a significant decrease in the thermo-mechanical properties.

The melting point of TLCP can also be greatly reduced by copolymerizing monomers with structures similar to those of HBA and HNA. A copolymer TLCP synthesized using a well-designed monomer is easy to process and has excellent physical properties, which can greatly expand its applicability. In addition, a thermotropic liquid crystalline copolyester (Co-TLCP) synthesized using various monomers will possess the necessary physical properties of the respective monomers^3,8,12,13^. Hence, Co-TLCPs of varying compositions, such as Xydar, Vectra, and X7G, have been extensively developed^3,14^.

Melt polymerization is a widely used technique, in which the monomer is melted at a high temperature using an extruder to enable polymerization. Because polymerization rapidly occurs in a single process, it is cost-effective and time-saving, yielding polymers with a higher molecular weight than those obtained from solution polymerization. However, it is difficult to precisely control the monomer amount in melt polymerization, which produces polydisperse polymers, i.e., wide molecular weight distribution. Further, melt-spinning is considered the most economical spinning process for polyester processing because the solvent is not recovered or evaporated and the spinning speed is relatively high, similar to dry-jet wet spinning. Thus, it is extensively used in the textile industry^5,7,15^.

Heat treatment, also called solid-state polymerization or annealing, is a well-established and widely used method. It is performed on polyesters and nylons obtained from conventional solution or melt polymerization. It generally increases the molecular weight and improves the physical properties of polymers such as poly(ethylene terephthalate) (PET) or nylon chips. This is because heat treating a polymer below its melting point affects the molecular structure, thereby increasing the crystallinity and improving the physical properties^16–19^. By improving their moisture-resistance and performance in extreme low-temperature environments through heat treatment, TLCPs can be used for various applications, including marine ropes, cables, fishing nets, sports nets, printed circuit boards, aerospace, and military composite materials that require extreme performance^20,21^.

In this study, Co-TLCPs were synthesized with melt polymerization using the monomers 2,5-diethoxy terephthalic acid (DTA), in which diethoxy groups are symmetrically substituted; 2,7-dihydroxynaphthalene (DHN), which exhibits a bent structure; and HBA. The amounts of DTA and DHN were kept constant while that of HBA was varied (0–5 mol), and their effects on the thermo-mechanical properties, thermal stability, liquid crystalline mesophase, and degree of crystallinity (DC) of the synthesized Co-TLCPs were determined.

To determine the effect of heat treatment, a melt-spun Co-TLCP fiber composed of 3 mol HBA, DTA, and DHN was used, and the effect of time and temperature (annealing conditions) on its thermo–mechanical properties was studied. In addition, the morphology of the fibers under various annealing conditions was investigated using scanning electron microscopy (SEM).

## Materials and methods

### Materials

Diethyl-2,5-dihydroxyterephthalate and hydrochloric acid (HCl) were purchased from Sigma Aldrich Chemical Co. (Yongin, Korea). DHN, HBA, acetic anhydride, and bromoethane were purchased from TCI Co. (Seoul, Korea), and anhydrous potassium carbonate (K_2_CO_3_), *N,N*′-dimethylformamide (DMF), and potassium hydroxide (KOH) were purchased from Daejung Co. (Seoul, Korea).

### Syntheses of the monomers

**Scheme 1:**
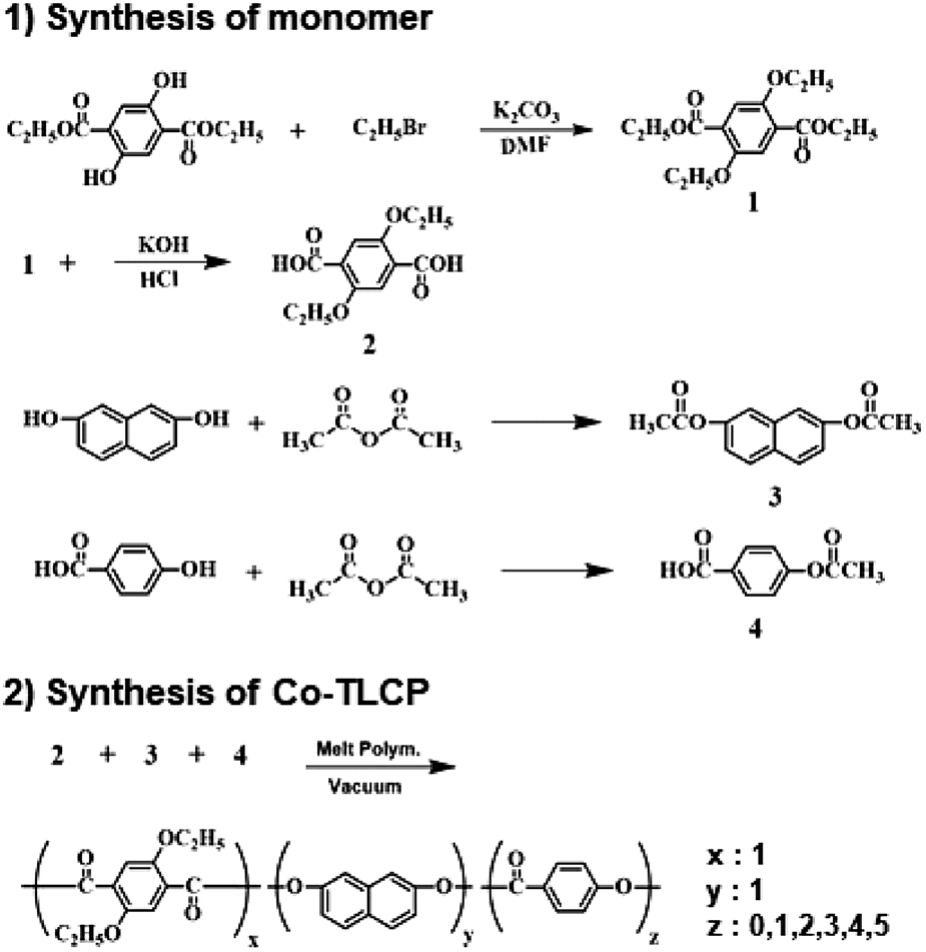
Synthetic routes for Co-TLCPs.

Diethyl-2,5-diethoxyterephthalate (monomer 1) and diethoxyterephthalic acid (monomer 2) were synthesized in several steps^22^. The acetylation reactions of DHN (monomer 3) and HBA (monomer 4) were completed using acetic anhydride. The detailed synthesis process is shown in Scheme 1.

### Syntheses of the Co-TLCPs

A Co-TLCP using the synthesized monomers 2–4 (Scheme 1) was synthesized by melt polymerization. The samples were named P-0, P-1, P-2, P-3, P-4, and P-5 to denote the HBA molar ratios of 0–5 mol, respectively (Table 1). As the synthesis methods of all Co-TLCPs were similar, only P-3 will be discussed as an example: Synthesized DTA (25.42 g; 1.0 × 10^−1^ mol), DHN (24.42 g; 1.0 × 10^−1^ mol), and HBA (54.05 g; 3.0 × 10^−1^ mol) were placed in a polymerization tube and vigorously stirred while gradually increasing the temperature under a uniform nitrogen stream. Acetic acid was generated in this process, and polymerization was completed at a constant reaction time under vacuum conditions to increase the molecular weight. The product (Co-TLCP) obtained in solid form was slowly cooled to room temperature, and any unreacted monomers and small molecules were removed by Soxhlet extraction with acetone for 48 h. The Co-TLCP was then dried in a vacuum oven at 60 °C for 24 h. Table 2 shows the detailed polymerization conditions for the synthesized TLCPs according to the HBA content. The intrinsic viscosities of the synthesized Co-TLCPs were measured in a mixed solvent of phenol/p-chlorophenol/TCE = 25/40/35 (w/w/w); however, their values were not obtained because the samples were completely insoluble in the solvent (see Table 3).

**Table 1:** Melt polymerization conditions of Co-TLCPs.

TLCPs	DTA^a^	DHN^b^	HBA^c^
P-0	1	1	0
P-1	1	1	1
P-2	1	1	2
P-3	1	1	3
P-4	1	1	4
P-5	1	1	5

^a^2,5-Diethoxyterephthalic acid (DTA).

^b^2,7-Dihydroxynaphthalene (DHN).

^c^4-Hydroxybenzoic acid (HBA).

**Table 2:** Melt polymerization conditions of Co-TLCPs.

Co-TLCPs	Temperature, °C/time, min/pressure, torr
P-0	250/60/760 → 270/50/760 → 280/30/760 → 290/50/760 → 290/30/240 → 290/50/1
P-1	250/40/760 → 270/120/760 → 270/60/240 → 270/40/1
P-2	250/30/760 → 270/30/760 → 280/30/760 → 290/80/760 → 290/30/240 → 290/50/1
P-3	245/120/760 → 250/30/760 → 265/50/760 → 270/50/240 → 285/40/1
P-4	250/120/760 → 265/50/760 → 280/60/760 → 285/50/240 → 290/30/1
P-5	260/60/760 → 270/60/760 → 280/60/760 → 295/30/760 → 295/30/240 → 295/30/1

**Table 3:** General properties of Co-TLCPs.

Co-TLCPs	IV^a^	T_g_ (℃)	T_f_^b^ (℃)	T_m_ (℃)	T_i_ (℃)	T_D_^ic^ (℃)	wt_R_^600d^ (%)	LC phase	DC^e^ (%)
P-0	N.O.^f^	130	200			360	31	No	0
P-1	N.O	132	200			370	41	No	0
P-2	N.O	130	200			352	38	No	3
P-3	N.O	107		278	305	323	36	Nem.^g^	15
P-4	N.O	113		287	311	339	38	Nem	16
P-5	N.O	113		311	343	369	41	Nem	18

^a^Inherent viscosity was measured at a concentration of 0.1 g/dL solution in a phenol/p-chloro-phenol/TCE = 25/40/35 (w/w/w) at 25 °C.

^b^Flow temperature observed by polarized optical micrographs.

^c^At a 2% initial weight-loss temperature.

^d^Weight percentage of residue at 600 °C.

^e^Degree of crystallinity.

^f^Not observed.

^g^Nematic.

### Extrusion

During spinning, approximately 20–30 g of the spinning sample was used at a time and the capillary rheometer maintained a temperature of 240–245 °C. The die diameter of the capillary rheometer was 0.50 mm, the average residence time in the die was approximately 2–3 min, and the spinning speed of the fiber was 15 m/min.

### Fiber heat treatment

Heat treatment is typically performed at a temperature between the glass transition temperature (*T*_*g*_) and melting transition temperature (*T*_*m*_) of a polymer. Under appropriate temperature and time conditions, a significant effect on the physical properties was observed. Thus, spinning was only performed on P-3, which exhibited a LCP and the lowest *T*_*m*_. This is because fibers can be easily and stably obtained without decomposition at high temperatures through melt spinning. Then, the melt-spun P-3 fibers were heat treated at different temperatures of 180, 210, and 240 °C and constant time of 3 h, and at a constant temperature of 240 °C and varying times of 6, 9, and 12 h.

### Characterization

To investigate the thermal properties of the synthesized Co-TLCP, differential scanning calorimetry (DSC; NETZSCH F3, Berlin, Germany) and thermogravimetric analysis (TGA; TA instruments Q500, New Castle, DE, USA) were performed in a nitrogen atmosphere at a ramp rate of 20 °C/min. The degree of crystallinity (DC) of TLCP was determined using wide-angle X-ray diffractometry (XRD; Rigaku (D/Max-IIIB, Tokyo, Japan) with a Cu-Kα (λ = 1.54 Å) target. Measurements were performed at room temperature at a rate of 2°/min. To observe the liquid crystalline behavior above the *T*_*m*_, a polarized optical microscope (POM) equipped with a hot stage (Mettler Toledo FP82HT, Tokyo, Japan) was used with a scanning speed of 5 °C/min. Measurements were obtained in the range of approximately 280–315 °C.

A universal testing machine (UTM) (Shimadzu AG-50kNX, Tokyo, Japan) was used to investigate the mechanical properties of the spun fibers at a cross-head speed of 20 mm/min. The experimental errors of the ultimate tensile strength and initial modulus were within ± 1 MPa and ± 0.05 GPa, respectively, while values with large error ranges were discarded. The results are an average of more than 10 measurements. To observe the fibrous structure of the heat-treated fiber, it was segmented by quenching in liquid nitrogen, and the fractured cross-section was analyzed using SEM (JEOL JSM-6500F, Tokyo, Japan).

## Results and discussion

### Thermal properties

The DSC results (Table 3) revealed that *T*_*g*_ was affected by segmental motion, which in turn was influenced by the size of the substituents attached to the main chain. When the main chain is bent and contains bulky substituents, the segmental motion of the main chain is disrupted and the *T*_*g*_ of TLCP increases^23,24^.

The *T*_*g*_ values of the synthesized Co-TLCPs were in the range of 107–132 °C depending on the HBA content. P-0, synthesized using only DTA and DHN monomers (without HBA), exhibited a *T*_*g*_ of 130 °C, which almost remained the same (130–132 °C) at 1 and 2 mol HBA. At 3 mol HBA, it decreased to 107 °C, but increased to 113 °C at 5 mol HBA. At a low HBA content, such as in P-3, the polymer structure is disrupted and the chain mobility increases, thereby lowering the *T*_*g*_ (107 °C). However, at a higher HBA content of 5 mol, HBA partially formed poly(hydroxy benzoate) (PHB), which is a block copolymer. This restricted the movement of the entire chain and *T*_*g*_ increased to 113 °C. In general, because block copolymers restrict chain movement, the thermal properties denoted by *T*_*g*_ improve. This phenomenon was also observed in Co-TLCPs composed of other monomers previously synthesized^11,18,25^.

A *T*_*m*_ peak was not observed for TLCPs containing 0–2 mol HBA because of the flexible alkyl group of DTA and the bent structure of DHN. Instead, a flow temperature (*T*_*f*_) was observed through POM at approximately 200 °C. However, at 3 mol HBA, a *T*_*m*_ was observed at 278 °C, which increased to 311 °C at 5 mol HBA. This increase in *T*_*m*_ can be attributed to the ease of molecular packing induced by the HBA monomer of the rigid rod-type^25–28^.

The isotropic temperature (*T*_*i*_) showed a similar trend to that of *T*_*m*_ (Table 3). An LCP was not observed for TLCPs with 0–2 mol HBA because of the bent structure of DHN and the flexible alkoxy side group of DTA. The *T*_*i*_ of the LCP depends on the segment length (σ_o_) and aspect ratio (σ_o_/d) of the physical hard-core thickness (d). A typical TLCP has a high aspect ratio of σ_o_/d ≥ 4^29^. The simple and linear structure of HBA enables the formation of an LCP at high concentrations only. However, when the HBA content increased from 3 to 5 mol, the *T*_*i*_ increased from 305 to 343 °C, which can be explained some of the HBA forms block copolymer when HBA was in excess, as previously described^25^ The detailed DSC results are shown in the Supplementary Information S1.

Table 3 summarizes the TGA results, which show that HBA content directly affects the 2% weight loss at the initial decomposition temperature (*T*_*D*_^*i*^). At 0 mol HBA (P-0), the *T*_*D*_^*i*^ was 360 °C, which rapidly decreased to 323 °C as the HBA content increased to 3 mol; note that P-1 (1 mol HBA) showed a higher *T*_*D*_^*i*^ (370 °C) than P-0 (0 mol HBA; 360 °C). At 3 mol HBA (P-3), the chain fluidity increased and *T*_*D*_^*i*^ decreased to 323 °C. At 5 mol HBA (P-5), the *T*_*D*_^*i*^ increased again to 369 °C because some chains formed block copolymers, as previously described. The residual weight at 600 °C (*wt*_*R*_^*600*^) of P-0 was 31%, lower than those of TLCPs with 1–5 mol HBA (36–41%). This indicates that the higher the aromatic content in the molecule, the higher the residual amount.

**Figure 1:**
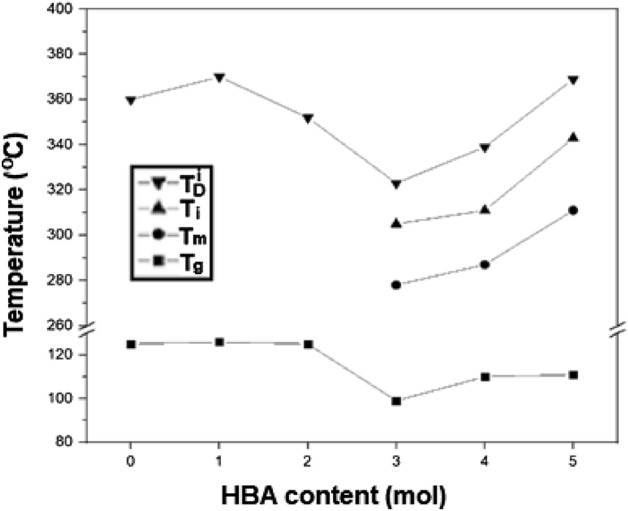
Thermal properties of Co-TLCPs with varying HBA content.

Figure 1 shows the effect of the HBA monomer concentration on the overall thermal properties (*T*_*g*_, *T*_*m*_, *T*_*i*_*,* and *T*_*D*_^*i*^) of Co-TLCP. *T*_*g*_ and *T*_*D*_^*i*^ showed the lowest values at 3 mol HBA, and the polymer exhibited eutectic behavior. The thermal properties improved when the HBA content was decreased or increased beyond 3 mol HBA, i.e., 3 mol HBA was the eutectic point (see Supplementary Information S1)^29–31^. The HBA monomer contributes toward improving the thermal properties of Co-TLCP; however, in excess, HBAs form some block copolymers. As a result, the thermal properties are compromised owing to the loose packing of the chains, and only stabilize at 1–3 mol HBA. However, beyond the eutectic point, the molecular packing tightens and crystallinity increases because of the appropriate HBA monomer concentration, thereby improving the thermal properties.

### Liquid crystalline mesophases

**Figure 2:**
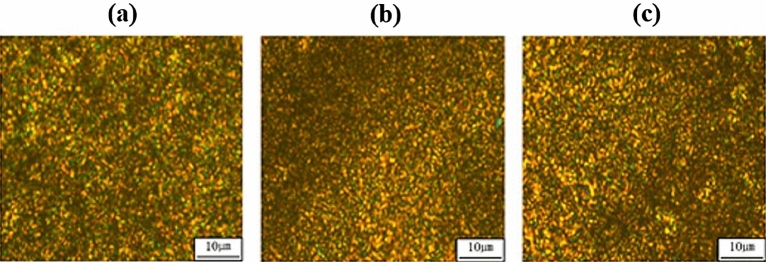
POMs of (**a**) P-3 at 280 °C, (**b**) P-4 at 295 °C, and (**c**) P-5 at 315 °C (magnification 200 ×).

Liquid crystalline mesophases were observed between *T*_*m*_ and *T*_*i*_ with the POM (Fig. 2); all the liquid crystalline mesophases exhibited thread-like schlieren nematic textures. The stability of the liquid crystal in Co-TLCP depends on the stiffness and aspect ratio of the mesogenic units^27,28^. As the content of HBA in Co-TLCP increased, the liquid crystal mesophase of the polymer main chain is stabilized.

A liquid crystalline mesophase was not observed in P-0, P-1, and P-2 because of the dialkoxy side group of DTA and the bent structure of DHN. In particular, the HBA content in P-1 and P-2 (1 and 2 mol HBA, respectively) was insufficient to form an LCP, while nematic liquid crystalline textures were observed in TLCPs with 3–5 mol HBA at 280, 295, and 315 °C, respectively (see Fig. 2).

### XRD of TLCP

Table 3 summarizes the DC obtained using XRD. P-0, P-1, and P-2 exhibited amorphous properties because of DTA and DHN, suggesting that these monomers are not suitable for increasing crystallinity. For P-3, P-4, and P-5, the DC increased by 15–18% as the HBA content increased. That is, it was confirmed that the structure of Co-TLCP changed from amorphous to semi-crystalline as the HBA content increased. The detailed results of XRD are shown in the Supplementary Information S3.

### Thermal properties of the annealed fibers

**Figure 3:**
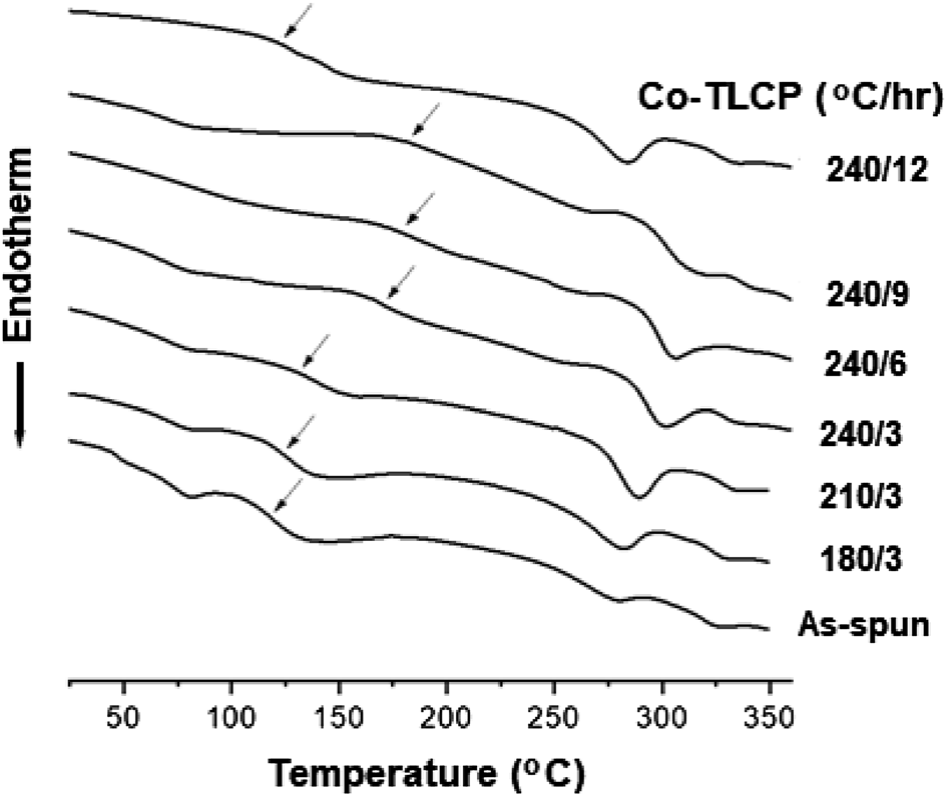
DSC thermograms of the heat-treated Co-TLCP fibers.

Among the various synthesized TLCPs, P-3 (3 mol HBA) fibers were selected for spinning. This is because P-3 showed liquid crystallinity, and had a low *T*_*m*_ and a stable liquid crystal range, which facilitates spinning (Table 3). The thermal properties of the fibers under various annealing conditions were investigated (Fig. 3 and Table 4).

**Table 4:** Thermal properties of the heat-treated Co-TLCP fibers.

Co-TLCP (°C/h)	T_g_ (°C)	T_m_ (°C)	T_i_ (°C)	T_D_^ia^ (°C)	wt_R_^600b^ (%)	DC^c^ (%)
As-spun	120	281	325	339	39	17
180/3	125	283	329	353	38	18
210/3	133	290	334	367	39	18
240/3	174	302	334	393	43	20
240/6	181	307	341	397	43	21
240/9	183	324	346	401	45	23
240/12	124	284	337	357	38	13

^a^At a 2% initial weight-loss temperature.

^b^Weight percent of residue at 600 °C.

^c^Degree of crystallinity.

In Fig. 3, a weak endothermic peak at approximately 80 °C appears until the heat treatment condition of 240/3, which may be attributed to the low molecular weight fraction generated during TLCP synthesis. This peak disappears as the heat treatment temperature and time are increased. The as-spun fiber demonstrated a *T*_*g*_ of 120 °C, which was slightly higher than that of P-3 in the powder form (107 °C). This can be attributed to the rigid amorphous fraction (RAF) formed during fiber spinning. After thermal annealing, the crystals gradually become tightly packed and the RAF becomes more rigid with increased *T*_*g*_^32,33^. This phenomenon was the same for all physical properties (*T*_*m*_, *T*_*i*_, *T*_*D*_^*i*^, *wt*_*R*_^*600*^, and DC) (Table 4).

Compared to the as-spun fiber, the *T*_*g*_ of the fiber at the annealing conditions of 180 °C and 3 h increased from 120 to 125 °C, followed by a significant increase to 174 °C at 240 °C and 3 h. It is known that *T*_*g*_ is largely dependent on the interactions between the polymer chains, and the segmental motion is closely related to the change in free volume. Heat treatment increases the *T*_*g*_ because it limits the segmental motion by improving the crystal packing and increasing the RAF in the fiber^23^.

The effect of constant temperature (240 °C) and varying times (3–9 h) was also investigated; the *T*_*g*_ increased from 174 to 183 °C with increasing annealing time. The effect of heat treatment on the *T*_*g*_ has been described above. Fibers obtained under 240/9 (240 °C and 9 h) annealing conditions showed the highest *T*_*g*_ value (183 °C) and were deemed as the optimum heat treatment conditions. The high *T*_*g*_ was attributed to the stiffness of the main chain; thus, the chain mobility became restricted. Heat treatment increases the crystallinity and molecular weight, which creates a high rotational barrier against segmental motion. However, at 240/12, *T*_*g*_ decreased by 59 °C (124 °C), which can be attributed to the deterioration caused by the extended exposure to excessive heat. An excessively harsh heat treatment also disrupts the molecular orientation or crystallization of the mesogen, which affects the segmental motion of the chain, thereby leading to a decrease in *T*_*g*_.

The effect of the annealing conditions on the *T*_*m*_ and *T*_*i*_ followed the same trend as that of the *T*_*g*_. The *T*_*m*_ of the as-spun fiber (without heat treatment) was 281 °C, which increased to 302 °C at 240/3 and to 324 °C at 240/9. These results indicate that the heat treatment induces changes in the crystal structure of the TLCP, i.e., an increase in the crystal size or improvement in the molecular packing due to an increase in molecular weight is observed^34,35^. However, at 240/12, the *T*_*m*_ sharply decreased to 284 °C because the crystallinity decreases as a result of the harsh heat treatment conditions and difficulty in mesogen packing (Table 4). The *T*_*i*_ of the as-spun fiber was 325 °C and demonstrated the highest value of 346 °C at 240/9, but decreased to 337 °C at 240/12. Similar to the trends observed for *T*_*g*_ and *T*_*m*_, *T*_*i*_ improved under the appropriate annealing conditions (240/9), but decreased above critical conditions^35^.

**Figure 4:**
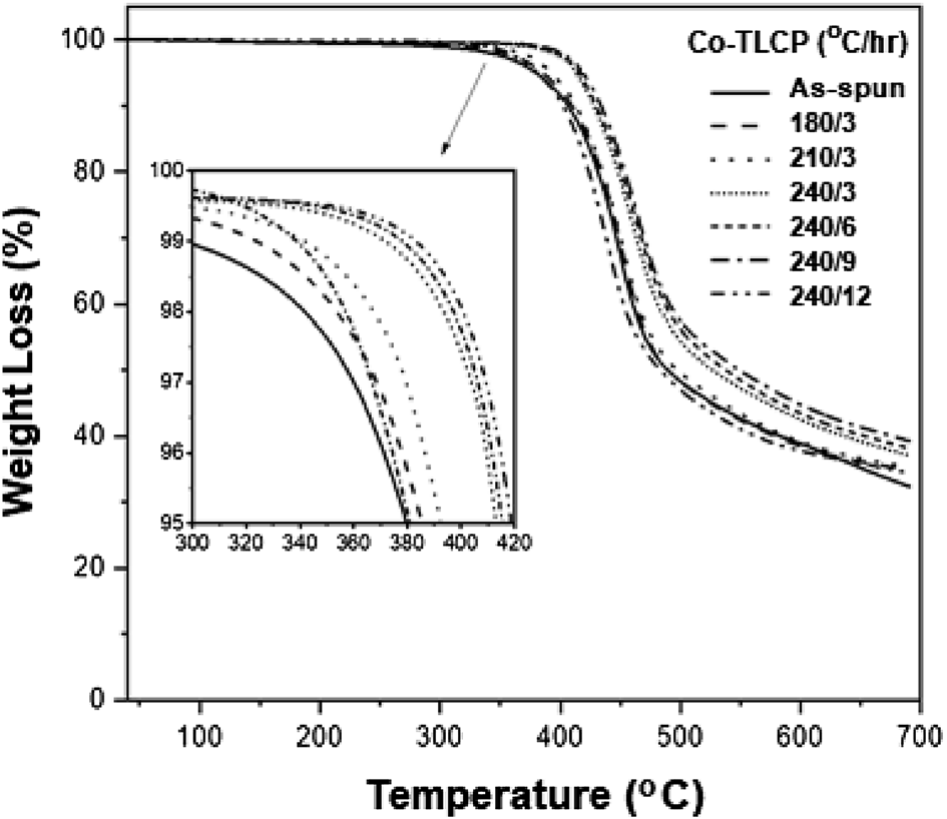
TGA thermograms of the heat-treated Co-TLCP fibers.

Comparing the as-spun fibers and those at 240/3, the *T*_*D*_^*i*^ also increased from 339 to 393 °C. It was highest (401 °C) at 240/9, but decreased to 357 °C at 240/12. The *wt*_*R*_^*600*^ also showed the same trend: a maximum value of 45% was observed at 240/9, which decreased to 38% at 240/12. Figure 4 shows the TGA results of the Co-TLCP fibers obtained under various heat treatment conditions, which have a significant effect on the thermal stability of the TLCP. This indicates that this process can be widely used to increase the thermal stability of TLCPs containing ester groups.

### XRD patterns of the annealed fibers

In general, as annealing increases the molecular weight and induces a fine crystal structure growth, the crystallinity of the fiber increases^36^. In particular, appropriate annealing conditions can be extremely effective in improving the physical properties of the fibers.

The DC of the as-spun fiber was 17%, which increased to 20% at 240/3. However, further increasing the annealing time (6–9 h) at 240 °C did not significantly improve DC (23%), but a sudden decrease (13%) was observed at 240/12 (Table 4). This is because the prolonged exposure to heat destroys the crystalline structure, as previously described.

**Figure 5:**
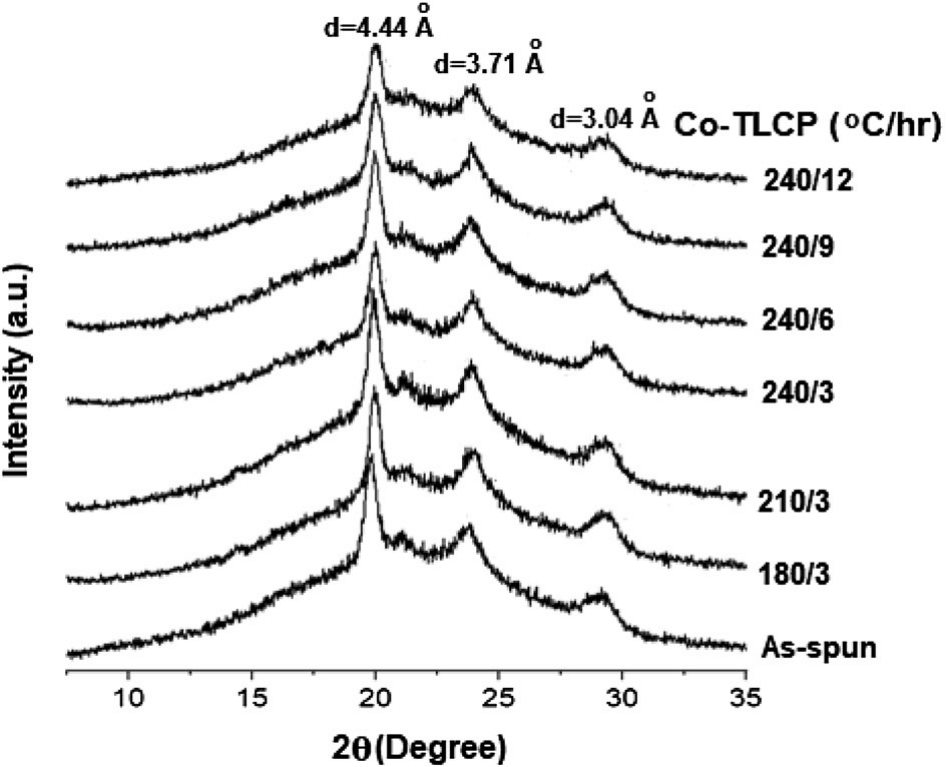
XRD patterns of the heat-treated Co-TLCP fibers.

Figure 5 summarizes the XRD patterns for the Co-TLCPs treated under various annealing conditions. Despite the varying annealing conditions, the same peaks were observed at a constant *2θ* position. These peaks gradually increased in intensity as the heat treatment time and temperature increased until 240/9, followed by a weakening at 240/12 at the same position.

### Mechanical properties of the annealed fibers

It is well known that heat treatment under appropriate conditions improves the mechanical properties of fibers^3,8,37^. As previously described in terms of thermal properties, when the RAF generated from the as-spun fiber is gradually increased under the heat treatment conditions, the mechanical properties improve because the intermolecular bonding or orientation between the polymer chains strengthens. The effect of the same annealing conditions on the mechanical properties, as those on the thermal properties, was determined (Table 5).

**Table 5:** The effects of heat treatment on the mechanical properties of Co-TLCP fibers.

Heat Treat. (Temp/time)	As-spun	180/3	210/3	240/3	240/6	240/9	240/12
Ultimate strength (MPa)	45	45	63	72	102	132	134
Initial modulus (GPa)	4.08	4.56	4.84	4.96	4.94	5.36	5.34
Elongation at break (%)	10	10	10	10	15	15	15

The ultimate tensile strength of the as-spun fiber was 45 MPa, which remained the same at 180/3. However, from 210/3 to 240/9, the tensile strength increased approximately three times (132 MPa) compared to the as-spun fiber, and only slightly increased to 134 MPa at 240/12. This trend has been previously described in terms of thermal properties. The initial modulus also exhibited a similar trend to that of the ultimate tensile strength. The initial modulus of the as-spun fiber was 4.08 GPa, which steadily increased to 5.36 GPa at 240/9 and then plateaued (5.34 GPa) at 240/12. The elongation at break (EB) was constant at 10% for the as-spun fiber and the fiber at 240/3. It increased to 15% at 240/9 and remained constant at 240/12 (Table 5).

In general, the polymer chains of commercialized TLCPs have a straight and rigid structure; therefore, they can successfully form anisotropic phases and are oriented in the same direction to form regular crystal structures. However, TLCPs containing semi-rigid or flexible chains have limitations in directionality because they are bent, folded, or entangled. Therefore, the flexible dialkoxy groups of DTA and the bent structure of DHN (specific to this study) interfered with the orientation, exhibiting poorer mechanical properties compared to wholly aromatic TLCPs. However, as mentioned in the introduction, TLCPs with these structures have a great advantage in that they are easy to process owing to their low *T*_*m*_.

### Morphologies of the annealed fibers

**Figure 6:**
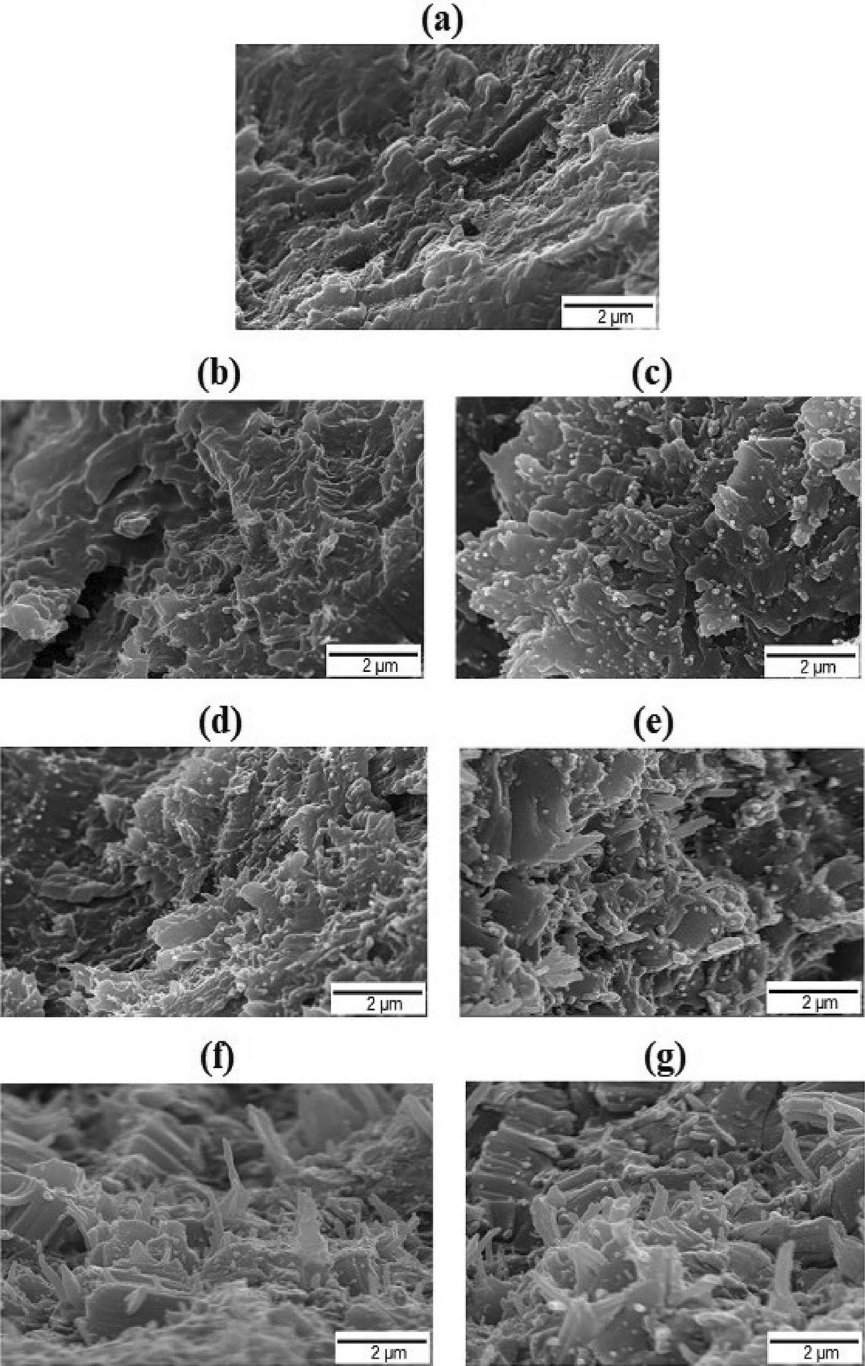
SEM images of the (**a**) as-spun, (**b**) 180/3, (**c**) 210/3, (**d**) 240/3, (e) 240/6, (**f**) 240/9, and (**g**) 240/12 °C/h heat-treated Co-TLCP fibers.

Fibers composed of general semi-crystalline polymers are a mixture of microfilaments and macrofilaments, and their boundaries are unclear^38^. To exhibit excellent thermal and mechanical properties, it is processed into fibers, followed by various post-treatment processes. In this study, the changes in the thermo-mechanical properties and DC obtained through the various heat treatments of fibers subjected to the spinning process were investigated. The annealed TLCP fibers were broken vertically in liquid nitrogen, and the cross-sections were observed using SEM (Fig. 6).

In the case of the as-spun fibers, an undeveloped morphology was observed in all areas (Fig. 6a). At 180/3, it was slightly more developed, but the fibrous form was not observed (Fig. 6b), indicating that this was not an ideal annealing condition for obtaining the required fiber formation. At 210/3, very small evenly distributed fibrous shapes were observed (Fig. 6c). The shapes became progressively defined with increasing annealing time (3–9 h) at 240 °C (Fig. 6d–f). In particular, at 240/9, fully grown evenly distributed fibrous formations were observed. This is because the RAF formed during fiber spinning gradually increases under suitable heat treatment conditions; thus, the TLCP fibers have a more improved crystal structure. This is also observed in general TLCPs or engineering plastic fibers treated to have excellent orientation, which further contributes toward the improved thermo–mechanical properties previously discussed. However, at 240/12 (Fig. 6g), the developed fibrous morphology did not significantly change compared to that at 240/9.

Overall, these results indicate that the fibers that were heat-treated at a relatively high temperature and longer time exhibited fine fiber shapes and were well-developed over the entire area, whereas those heat-treated at a low temperature and shorter time did not exhibit a fiber shape. However, extreme heat treatment conditions did not significantly improve the fiber formation.

**Figure 7:**
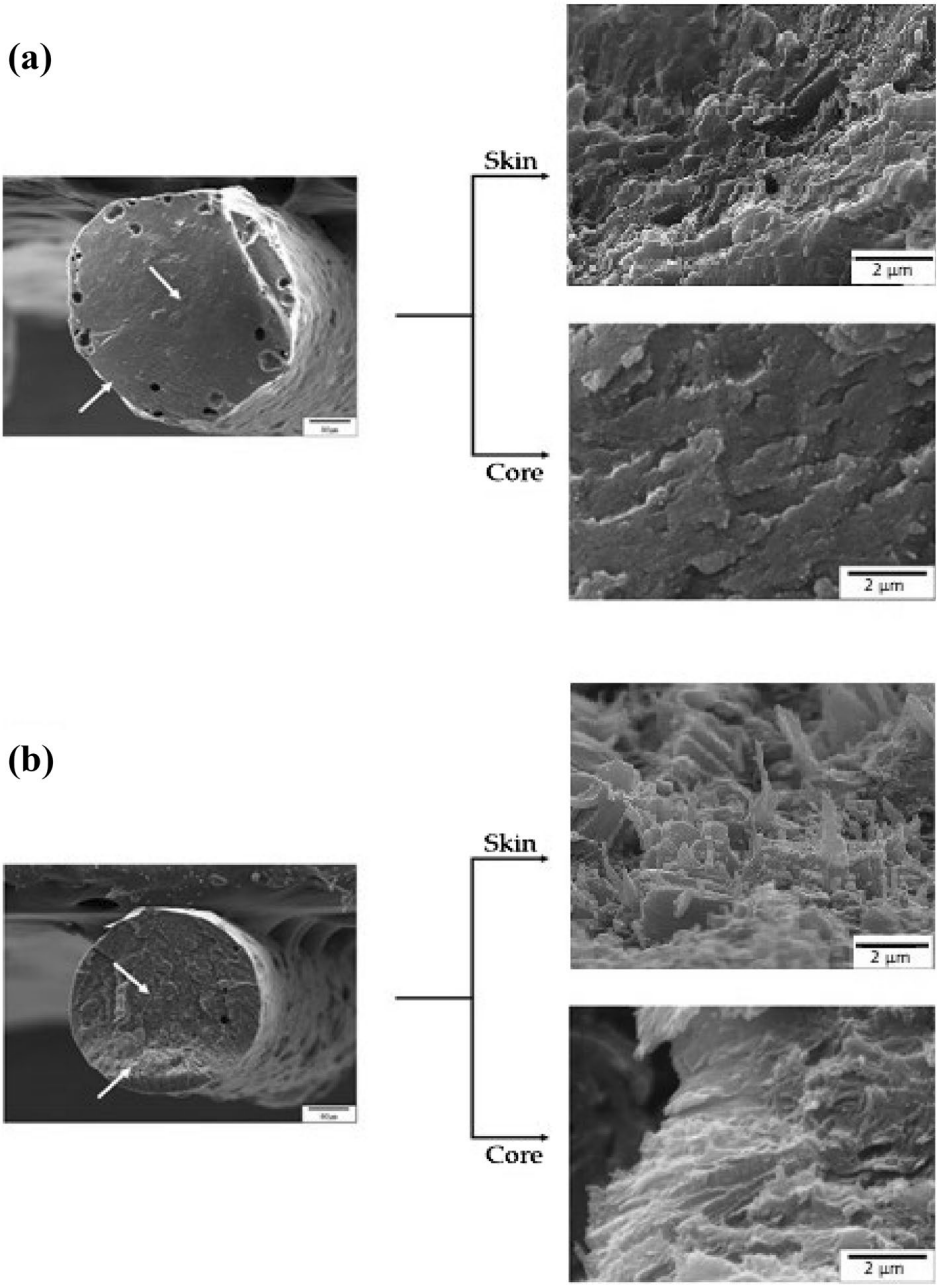
SEM images of the skin–core morphology in the (**a**) as-spun and (**b**) 240/9 fibers.

The molecular structure of TLCPs is mainly composed of straight and rigid rod-shaped mesogens. Therefore, when TLCPs are subjected to high heat and pressure during spinning in a rheometer, they exhibit a “skin–core” morphology, denoting a different molecular orientation at the center and the outer layer^39–42^. In the skin region of the fibers, heat and pressure directly affect the orientation of the polymer chains in the same direction as the flow, whereas in the core region, which is not directly affected by the heat and pressure, the molecular orientation of the polymer chains cannot be maintained in the parallel direction. This has been reported not only for TLCP, but also for common engineering plastics, such as polyester and nylon 6. Figure 7 shows the SEM micrographs of the as-spun fibers and those obtained under the 240/9 heat treatment condition. In the case of the as-spun fibers, there was no significant difference in the skin and core areas (Fig. 7a), suggesting that there was almost no difference in the orientation of the polymer chains in the skin and core. This is because of the mesogenic structure composed of DTA containing the dialkoxy groups and the bent structure of DHN. However, in the 240/9 fiber, which exhibited a higher DC, a difference in fiber orientation was observed owing to the heat and pressure conditions during spinning. That is, several developed fine fibers were observed in the skin area, but aggregates or non-directional fibers were observed in the core area (Fig. 7b). However, the fibrous forms observed via SEM were not highly developed and abundant compared to other TLCPs because the mesogen constituting the main chain contained flexible alkoxy groups or was composed of monomers with a bent structure.

**Figure 8:**
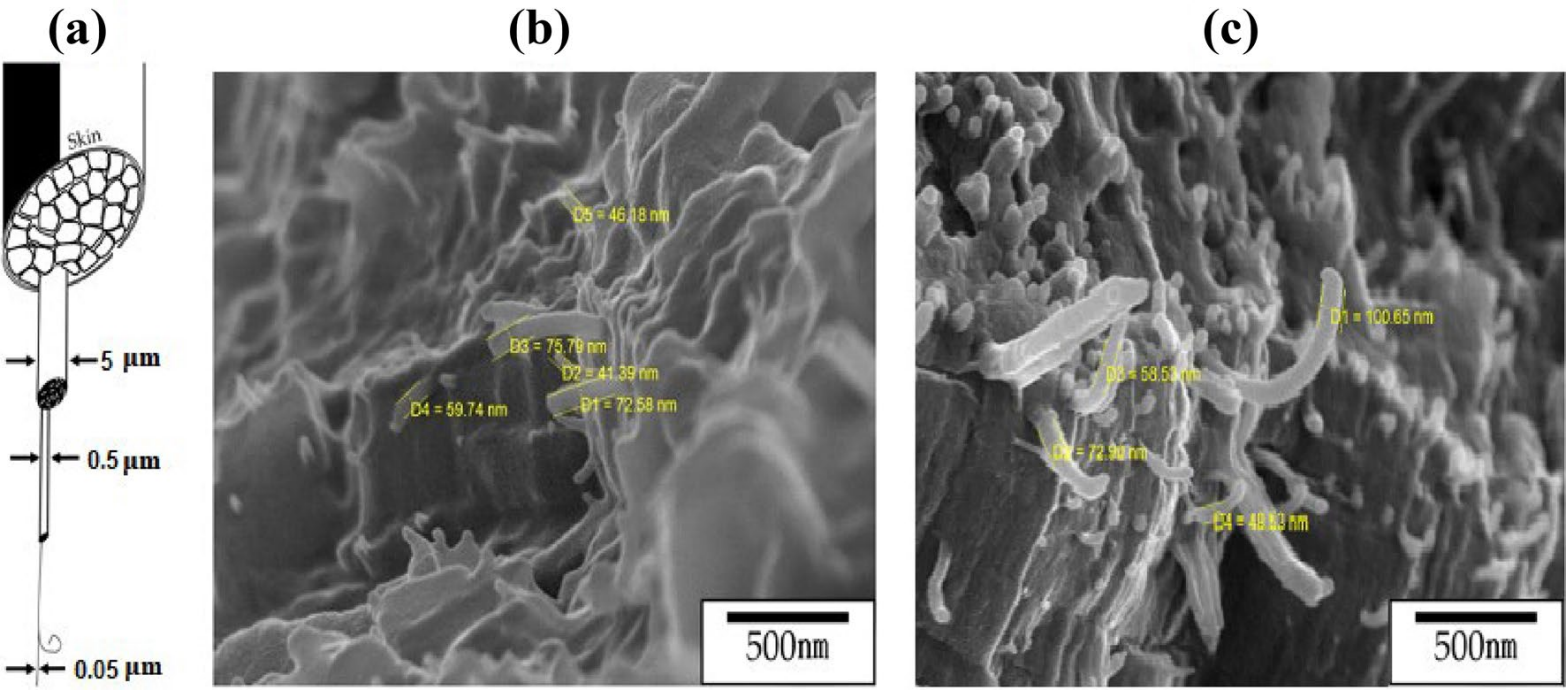
(**a**) Hierarchical structures in the Co-TLCP fibers^43^ and SEM images of the (**b**) 240/9 and (**c**) 240/12 annealed fibers.

In some cases, TLCPs exhibit highly-developed nanoscale fiber morphology owing to their rigid rod-shaped mesogenic structure. The hierarchical model of TLCP fibers by Sawyer et al.^43^ proposes that the basic fiber is an ultrafine fiber, several of which twist together to form a larger fiber bundle (Fig. 8a). In the well-oriented skin region of TLCP, a fiber bundle composed of several microfibers demonstrated a diameter of approximately 5 μm. However, upon gradually reducing the number of the fiber strands, a single ultrafine fiber of approximately 50 nm (0.05 μm) in diameter is defined. As a result, the TLCP fibers composed of several ultrafine fibers demonstrated excellent thermo–mechanical properties. To confirm the ultrafine fibers, the diameters of the fibers obtained under the 240/9 heat treatment condition were determined. These were in the range of approximately 41–76 nm (Fig. 8b). The values of the fibers under other conditions fall within this range as well. Figure 8c shows fibers with a diameter range of 48–100 nm obtained under the 240/12 condition, which were thicker than those obtained at 240/9. The detailed results of SEM are shown in the Supplementary Information S4.

## Conclusions

Co-TLCPs containing DTA, DHN, and HBA were synthesized using melt polymerization. For 0–2 mol HBA, *T*_*m*_, *T*_*i*_, and liquid crystalline mesophases were not observed because of the flexible dialkoxy group of DTA and the bent structure of DHN. At 3 mol HBA, all thermal properties of Co-TLCP (*T*_*g*_ and *T*_*D*_^*i*^) demonstrated minimum values, indicating a eutectic point, beyond which the thermal properties of Co-TLCP changed.

All thermal properties of the spun fibers gradually increased with increasing annealing temperature and time. The best properties were observed at 240 °C and 9 h and decreased under harsher annealing conditions. The mechanical properties of the as-spun fibers obtained by spinning were similar to those of general engineering plastics. Moreover, similar to the results of the thermal properties, all mechanical properties of the heat-treated fibers showed the best properties under the 240/9 annealing condition, and did not significantly change under harsher heat treatment conditions.

The cross-sections of the fibers produced during the spinning process revealed fine fibers under various annealing conditions. The higher the temperature and the longer the time during annealing, the finer the fibers produced. A skin–core morphology was also observed in the fiber zone when exposed to different heat and pressure conditions during spinning. The diameters of the spun ultrafine fibers ranged from approximately 40–100 nm, and these values were also observed for fibers obtained under other annealing conditions.

These findings reveal that proper heat treatment conditions play a significant role in improving the thermo-mechanical properties of TLCP fibers. As the heat treatment temperature and time increased, highly well-developed fibrous filaments were observed, while simultaneously increasing the size and number of the microcrystals, thereby improving the physical properties. However, harsher heat treatment conditions compromised these properties.

## Supplementary Information

Supplementary Figures.

Supplementary Figures.

## Data Availability

The datasets generated and/or analyzed during the current study are available from the corresponding author on reasonable request.
