# Supplementary material for: Annealing effect of thermotropic liquid crystalline copolyester fibers on thermo-mechanical properties and morphology
Source: Sci Rep. 2022 Jul 30;12:13100. doi: 10.1038/s41598-022-17431-5 (PMC9338994; doi:10.1038/s41598-022-17431-5)
Supplement: Supplementary file 1 — Supplementary Figures. [file 41598_2022_17431_MOESM1_ESM.docx]

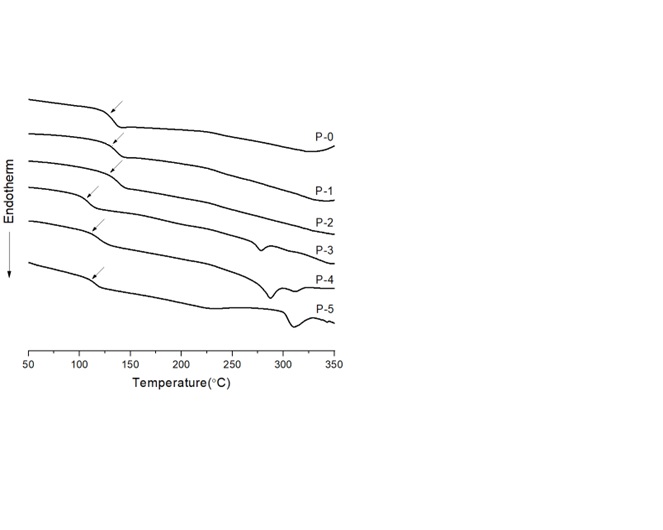


**Supplement - Figure S1.** DSC thermograms of Co-TLCPs according to various HQ contents.


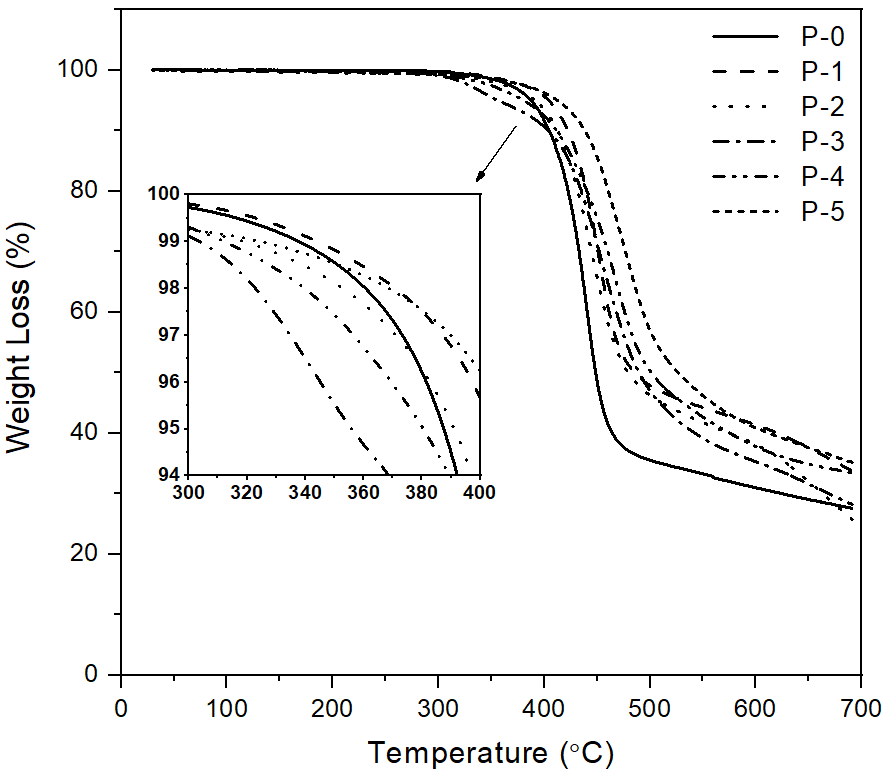


**Supplement - Figure S2.** TGA thermograms of Co-TLCPs according to various HQ contents.


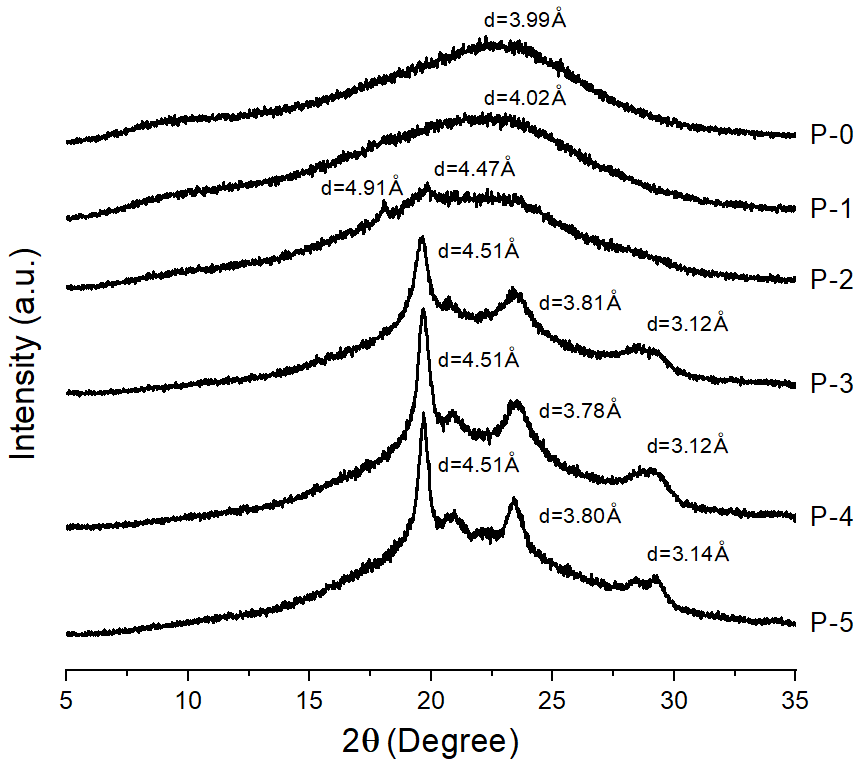


**Supplement - Figure S3.** XRD patterns of Co-TLCPs according to various HQ contents.
