# Supplementary material for: Annealing effect of thermotropic liquid crystalline copolyester fibers on thermo-mechanical properties and morphology
Source: Sci Rep. 2022 Jul 30;12:13100. doi: 10.1038/s41598-022-17431-5 (PMC9338994; doi:10.1038/s41598-022-17431-5)
Supplement: Supplementary file 2 — Supplementary Figures. [file 41598_2022_17431_MOESM2_ESM.docx]

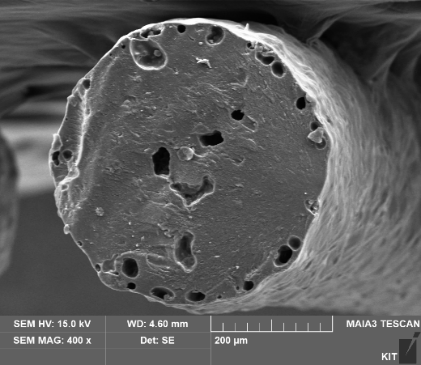


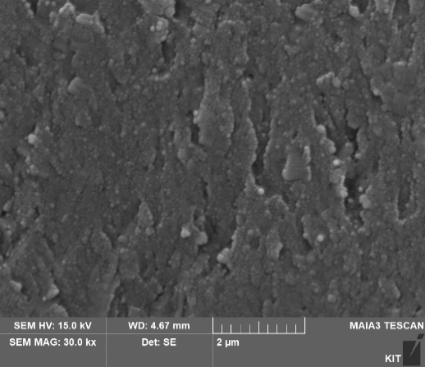

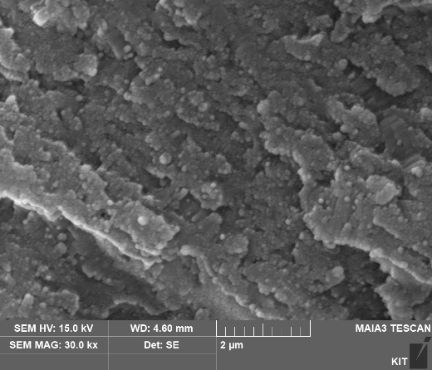

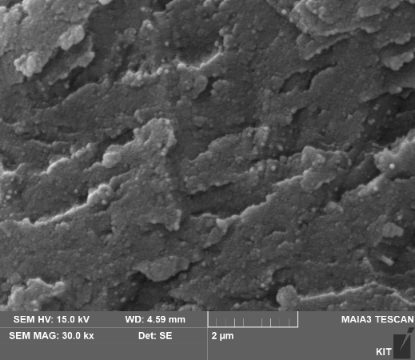


**Supplement – Figure S4 (a).** SEM photographs of As-spun TLCP fiber.


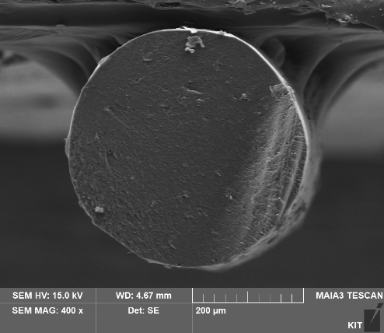


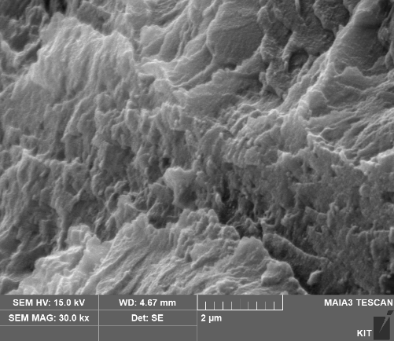

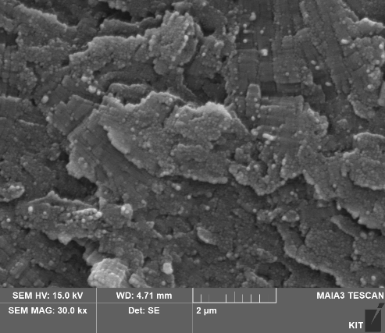


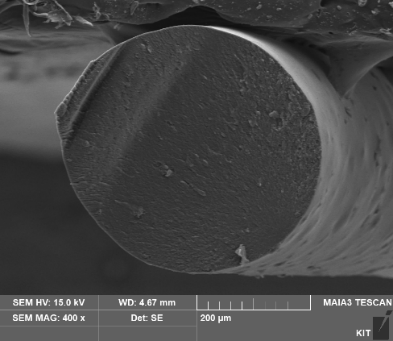


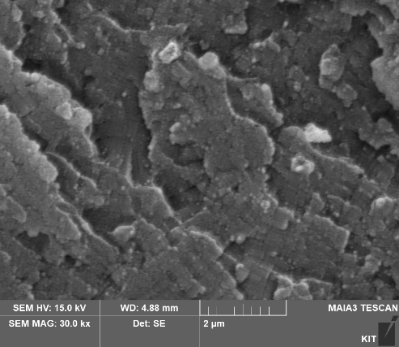

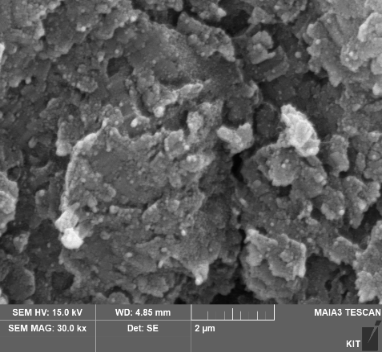


**Supplement – Figure S4 (b).** SEM photographs of 180/3 ^o^C/h TLCP fiber.


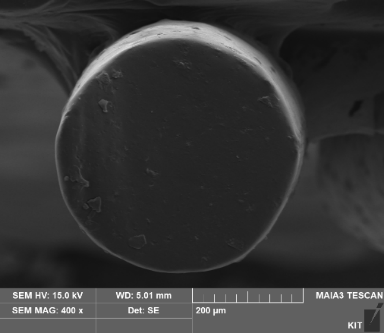


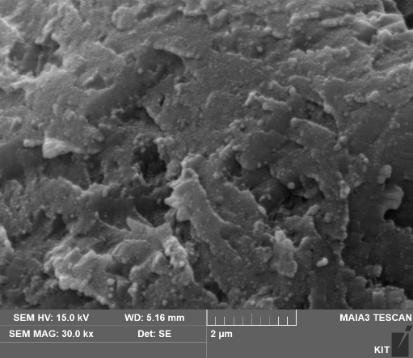

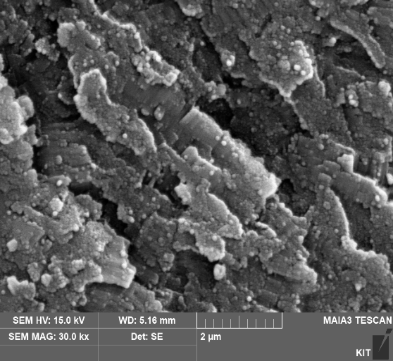

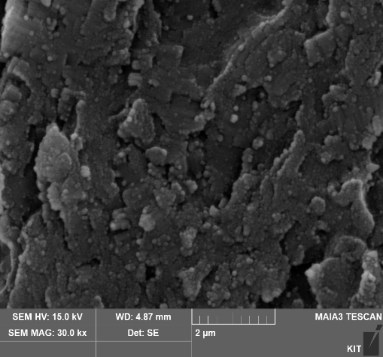


**Supplement – Figure S4 (c).** SEM photographs of 210/3 ^o^C/h TLCP fiber.


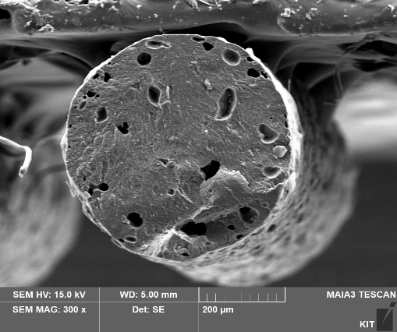


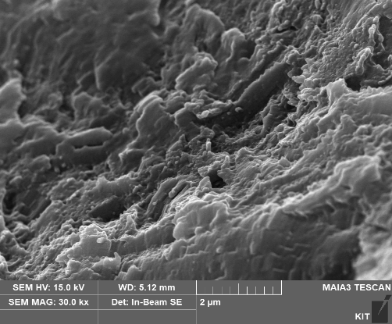

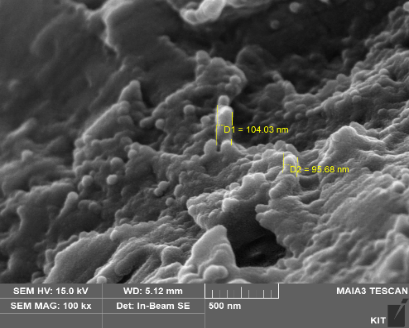


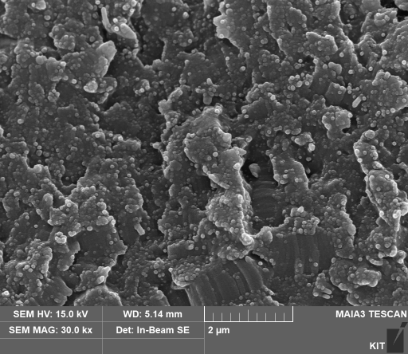

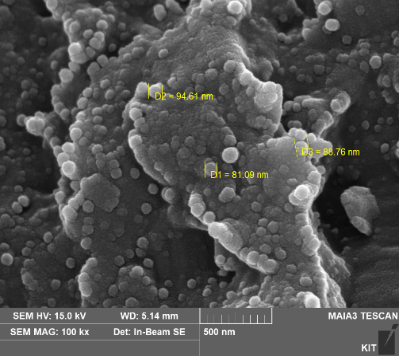


**Supplement – Figure S4 (d).** SEM photographs of 240/3 ^o^C/h TLCP fiber.


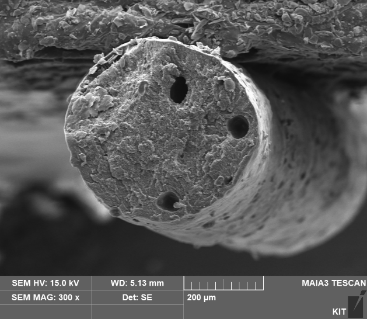


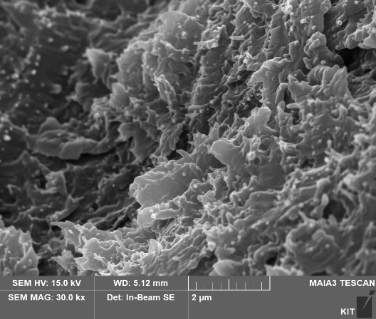

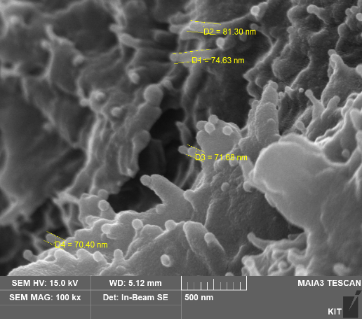

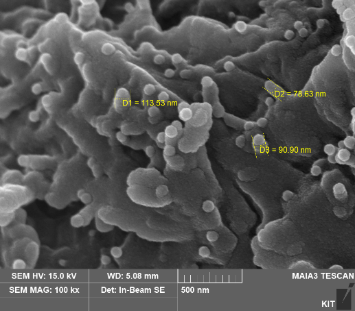


**Supplement – Figure S4 (e).** SEM photographs of 240/6 ^o^C/h TLCP fiber.


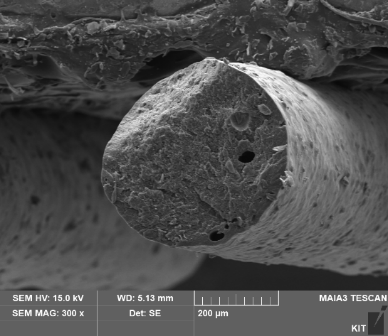


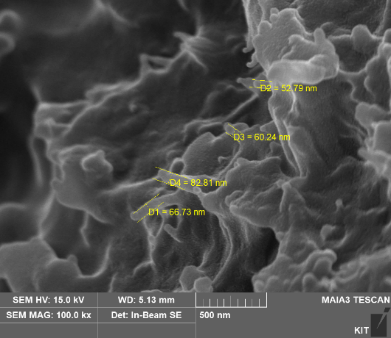

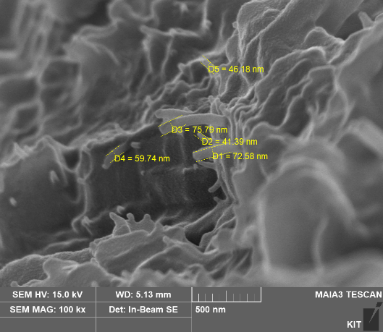

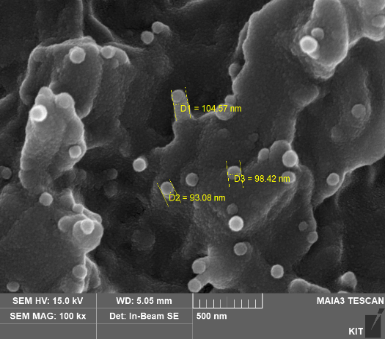


**Supplement – Figure S4 (f).** SEM photographs of 240/9 ^o^C/h TLCP fiber.


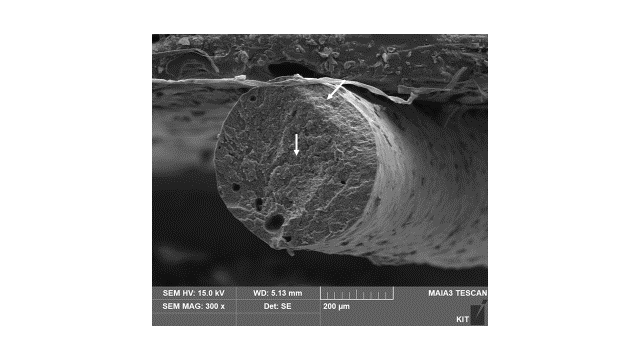


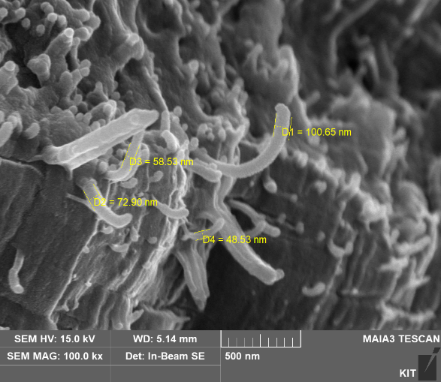

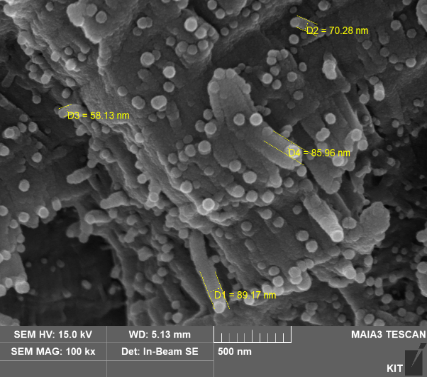


**Supplement – Figure S4 (g).** SEM photographs of 240/12 ^o^C/h TLCP fiber.
